# Heavy Metals, Gut Microbiota, and Biochemical Markers: Unraveling the Complexities of Obesity

**DOI:** 10.1002/mbo3.70071

**Published:** 2025-10-21

**Authors:** Ahmed Basim Mohamed Alamer, Majid Komijani, Shahnaz Shahrjerdi

**Affiliations:** ^1^ Department of Biology, Faculty of Science Arak University Arak Iran; ^2^ Department of Physiology and Sports Pathology, Faculty of Sport Sciences Arak University Arak Iran

**Keywords:** gut microbiome, heavy metals, obesity

## Abstract

Obesity is a global public health threat. This study investigated relationships between heavy metal exposure, gut microbiome composition, and obesity by comparing obese individuals and healthy controls. Heavy metal levels in stool were assessed via ICP‐MS, microbiome profiling via 16S rRNA sequencing, and biochemical parameters from blood. Key results showed obese individuals had significantly elevated FBS, cholesterol, triglycerides, LDL, VLDL, liver enzymes, and lymphocytes (*p* < 0.05). ICP‐MS revealed higher stool concentrations of Cd, Zn, Fe, Mn, and P, and lower levels of Ba, V, W, Ti, Ge, Nd, and S in obese subjects (*p* < 0.05). Although *Bifidobacteriaceae*, *Ruminococcaceae*, and *Coriobacteriaceae* were abundant across all groups with no significant difference in abundance (*p* > 0.05) or alpha diversity (*p* = 0.3), beta diversity revealed significant phylogenetic differences between controls and obese/lean groups (*p* < 0.0001). Specific correlations were identified: *Bifidobacteriaceae* abundance negatively correlated with cadmium (*r* = −0.6629, *p* = 0.0051) and fasting blood glucose (*r* = −0.61, *p* = 0.021). BMI positively correlated with Bacteroides abundance (*r* = 0.5851, *p* = 0.0190) and negatively with HDL (*r* = −0.68, *p* = 0.007). Iron negatively correlated with total cholesterol (*r* = −0.62, *p* = 0.019). *Coriobacteriaceae* abundance positively correlated with manganese (*r* = 0.55, *p* = 0.040) and ALT (*r* = 0.56, *p* = 0.039). *Ruminococcaceae* negatively correlated with triglyceride and VLDL (*r* = −0.55, *p* = 0.041). These findings suggest elevated heavy metal excretion links environmental exposure to obesity. Phylogenetic microbial differences, despite similar abundance, highlight environmental influences on gut microbiota, underscoring the importance of investigating environmental factors in metabolic health.

## Graphical abstract

Elevated excretion of heavy metals in obese individuals is correlated with specific gut microbiome shifts and worsened metabolic health, suggesting environmental toxins may contribute to obesity by altering gut ecology.

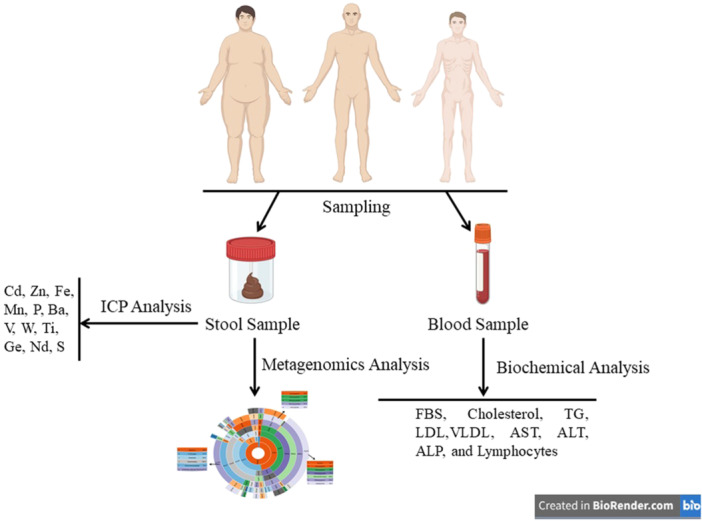

## 1 Introduction

Obesity is a fast‐growing public health threat on a worldwide scale, which is associated with significant health problems as well as a significant psychosocial burden for many individuals (Sarwer et al. 2012). Central mechanisms, such as disturbances in the homeostatic inhibition of food intake and lack of reward, and environmental agents, such as disturbances in nutritional feedback and neuro‐endocrine signaling, are considered essential factors in obesity. Of course, the physiology of an obese person, acquired pathophysiology, and genetics of energy metabolism have also been effective in obesity (Oussaada et al. 2019). The findings of several decades have shown that exposure to environmental pollutants may play an essential role in causing obesity. Their significant effect is disrupting the endocrine glands, and they are called “obesogenic” (Trasande and Blumberg 2018). In vitro investigations showed both up‐ and downregulation of adipogenesis associated with an unusual expression of critical adipogenic pathways, namely C/EBP and PPARγ. A comparison of the current analyses based on dose and way of exposure showed that the consequences of heavy metal exposure on fatty tissue might be dose‐dependent, ranging from increased adipogenesis at low‐dose exposure to inhibition of adipose tissue differentiation at higher doses (Tinkov et al. 2021). Recently, researchers have emphasized the increasing importance of gut microbiota in obesity. Gut microbiota changes affect host energy balance, energy production from the diet, and host genes regulating energy consumption and storage (Davis 2016). Changing the composition of the intestinal microbiota can disturb the homeostasis of human health and lead to the occurrence of metabolic diseases (obesity, diabetes, NAFLD) and cardiovascular diseases, etc (Fan and Pedersen 2021). The composition of the gut can be influenced by several dietary components, such as probiotics, including fermented foods, or prebiotics, such as inulin, other oligosaccharides, lactulose, and resistant starch, suggesting the possibility of manipulation of the microbiota. The gut may promote weight loss or prevent obesity in humans (Davis 2016). The aims of this thesis are to measure the level of Blood Glucose, Cholesterol, HDL, LDL, VLDL, and Triglyceride in the serum of severely obese patients; To analyze some heavy metals in their stool samples based on ICP MS ICP‐MS (Inductively Coupled Plasma Spectroscopy) method and compare them with healthy people to investigate the relationship between exposure to environmental pollutants such as heavy metals and the increased risk of obesity; To examine the effect of the gut microbiome on obesity and compare the gut microbiome of severely obese patients with the healthy group based on the 16S rRNA gene sequencing.

## 2 Materials and Methods

### 2.1 Collection and Preparation of Samples

The sampling was done between December 2022 and April 2023 from individuals admitted to Medical and health centers in Arak. The studies of patients with type 1 diabetes, defective glucose tolerance, metabolic syndrome, maturity‐onset diabetes of youth, and gestational diabetes were excluded. Obesity was defined as BMI ≥ 25 kg/m^2^. The Obese patient group was separated from the control and lean groups based on the differences in BMI.

### 2.2 Biochemical Assays: FBS, Lipid Profile, and Liver Enzyme Assays

The Cayman kit (Cat No:10009582) was used to measure the glucose. The cholesterol, Triglyceride, and HDL concentration (mg/dl) were determined using an automated analyzer (Biotecnica, BT 1500, Rome, Italy) and the commercial CHOD Pars Azmon kits (Cat No: 110 500, Cat No 132 504 H917, and Cat NO 1150 011, respectively.

The Randox/England AL101, and AL100 were employed for the quantitative in vitro assessment of Aspartate Aminotransferase (AST) and Alanine Aminotransferase (ALT) respectively, utilizing the UV method. This method is an optimized standard procedure aligned with concentrations recommended by the International Federation of Clinical Chemistry (IFCC), a widely recognized federation in the field (Bergmeyer et al. 1986). The diagnostic kit employed for the AP test was sourced from DiaSys in Germany (Cat No: 10401 9910021). The AP test followed a kinetic photometric approach, optimized based on the standard method outlined by the German Society of Clinical Chemistry (DGKC) (Deutsche 1972). All of the FBS, Liver profile and Liver enzyme assays were performed according to the manufacturers' protocol.

### 2.3 ICP‐MS Analysis

#### 2.3.1 Stool Sample Preparation

Each stool sample underwent filtration and assessment via 0.22 μm syringe filters. The wet digestion was used for sample processing and eliminating the impurities (Tang et al. 2016). The wet digestion method using Nitric acid (HNO3) and Perchloric acid (HClO4) was performed according to the protocol by Zhao et al 1994 to ready samples for analysis using ICP‐MS (Zhao et al. 1994). Following the preliminary sample preparation, the elemental analysis was carried out utilizing ICP‐MS (Thermo Scientific, iCAP RQ) equipped with an ASX‐280 autosampler for ICP‐MS. The analysis was conducted with the following parameters: **(**Carrier gas flow rate: 0.80 L/min; Auxiliary gas flow rate: 0.75 L/min; Cooling air flow rate: 13 L/min; Radiofrequency power: 13 kW) (Tang et al. 2016; Zhang et al. 2010).

### 2.4 16S rRNA Sequencing of Gut Bacteria

#### 2.4.1 DNA Extraction From Stool Samples

To isolate DNA from stool samples, we employed the Invitek's PSPStool DNA extraction kit (Cat No: 1038120200). The quantity of the extracted DNA was determined using nanodrop spectrophotometry. Additionally, the quality of the extracted DNA was assessed through agarose gel electrophoresis to observe the DNA band patterns. Upon verifying the DNA concentration, the samples were meticulously preserved in a freezer, maintained within the range of −20°C to °C80°C, in preparation for subsequent steps.

#### 2.4.2 Library Preparation

The DNA library was meticulously crafted through a two‐step PCR amplification method. The initial PCR phase amplified the specific region of interest, namely the 16S V3‐V4 region. Subsequently, during the second PCR, adapters and barcodes were introduced to these amplicons. The second round of PCR aimed to incorporate adaptors into the initial PCR products. This process involved an additional 5–10 cycles of PCR. Barcoded primers were diluted to a concentration of 2 µM, with each well of the 96‐well plate containing both the forward (F) and reverse (R) primers for the second PCR. During the second‐step PCR, barcodes were introduced to enable the simultaneous sequencing of multiple samples within a single sequencing run (Seitz et al. 2015). These target‐specific primers encompass universal sequences, which, during the second PCR, facilitated the extension of the amplicons with Illumina adapters and sample‐identification barcodes. The primers used in the first and the second rounds of PCR are presented in the Table S1.

#### 2.4.3 Quantity Analysis by Nanodrop Spectrophotometer

To achieve the highest DNA recovery possible for accurate and precise sequencing analysis (Shahi et al. 2019), both PCR products were quantified using a Nanodrop spectrophotometer.

### 2.5 Statistical Analysis

The data are presented as mean ± standard deviation. Normality was assessed using the Shapiro–Wilks test. Group comparisons were conducted using one‐way analysis of variance (ANOVA) with Tukey's multiple comparison post hoc tests for parametric data. For non‐parametric analysis (Kruskal–Wallis test), Dunn's multiple comparisons test was employed. Bivariate Spearman correlation was employed to assess the association between two quantitative variables. Logarithmic transformation was applied to certain variables with a non‐normal distribution. Statistical analyses were performed using GraphPad Prism 9.0, and significance was set at *α* = 0.05.

## 3 Result

### 3.1 Biochemical Assays

#### 3.1.1 BMI and FBS Assays

**Figure 1:**
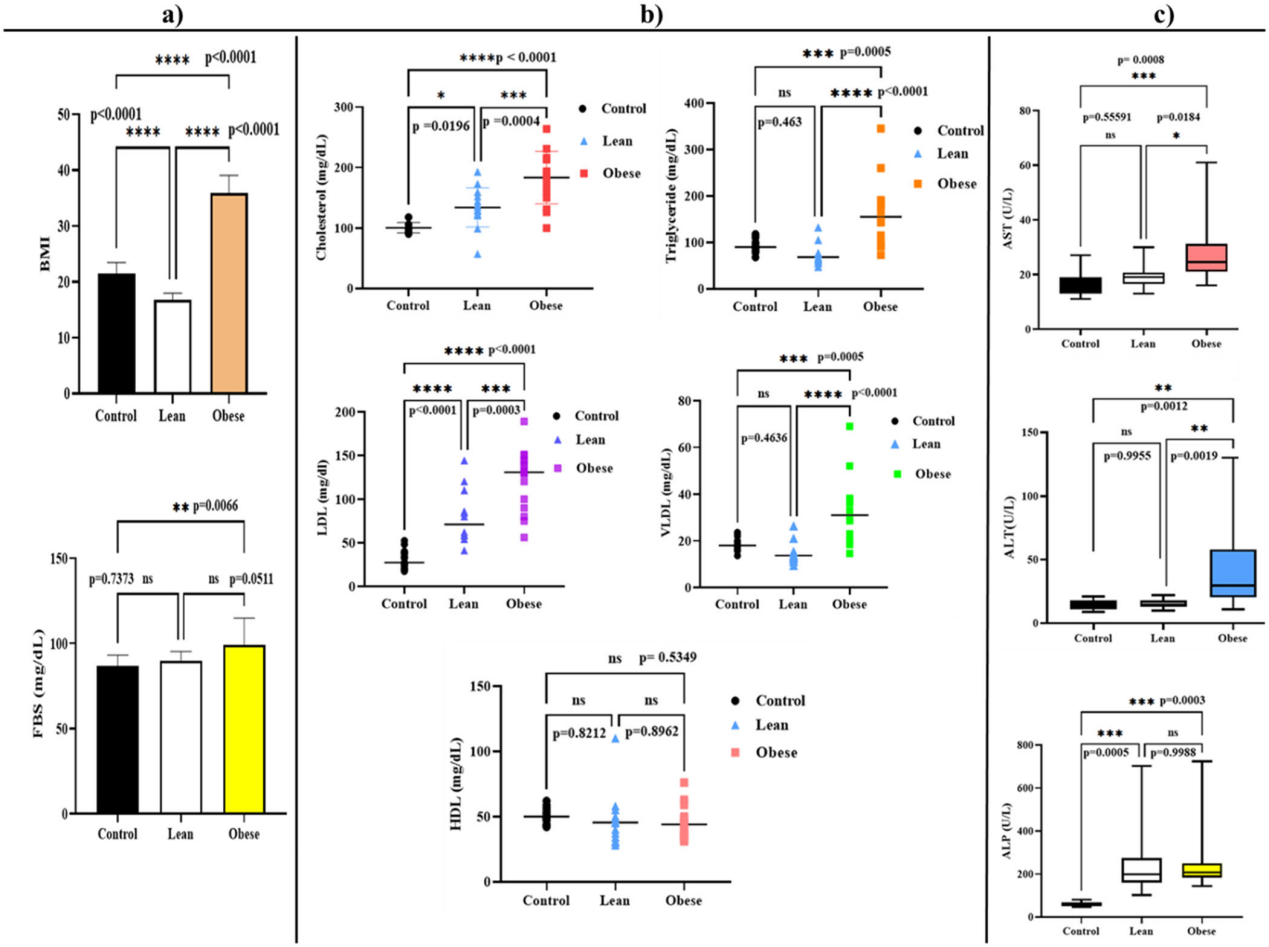
Comparison of the BMI, FBS levels. (a) Lipid profile assay (b) and liver enzymatic assays (AST, ALT, ALP), (c) between the control, lean, and obese groups.

The enrolled individuals in this study were classified into three groups according to their BMI: the Control group with a normal BMI (20–25 kg/m^2^), the lean group (< 20 kg/m^2^), and the Obese group with a BMI more than 25 kg/m^2^. The BMI of all individuals was calculated, and the glucose level in the bloodstream following a fasting period was assessed. The One‐way ANOVA test was employed to compare the differences in BMI and FBS assays between the control, Lean, and obesity groups. The findings indicated a significant elevation in FBS levels in the obesity group compared to the control group (*p*‐value of 0.0066 **), as illustrated in Figure 1a.

#### 3.1.2 Lipid Profile Assay

The results of the lipid profile assay are shown in the Figure 1b. Along with One‐way ANOVA, and also the Tukey's post‐hoc test was used to multiple comparisons between the mean of all possible pairs. The comparison between groups revealed a significantly higher concentration of Cholesterol, TG, LDL, and VLDL in the obese individuals compared to the normal (Control) and Lean groups. For HDL assay, no significant difference was observed between the HDL levels in the three studied groups (Control vs. Obese (*p* = 0.5349) and Lean vs. Obese *p* = 0.8962) (Figure 1b).

#### 3.1.3 Liver Enzyme Assay

The enzymatic activity of the liver, encompassing the measurement of three enzymes, was evaluated in three groups. As depicted in Figure 1c, the statistical analysis revealed a higher activity of the enzymes AST and ALT in the obese group compared to the control and Lean groups. In the case of the ALP activity, the result showed significantly higher activity in the obese and lean groups compared to the control group. In addition, as depicted in Figure 1c, no significant difference was shown between the obese and the lean groups (*p* = 0.9988).

### 3.2 ICP‐Analysis: Significantly Higher Levels of Cd, Zn, Fe, Mn, P in the Feces Samples of the Individuals With Abnormal Weight

**Figure 2:**
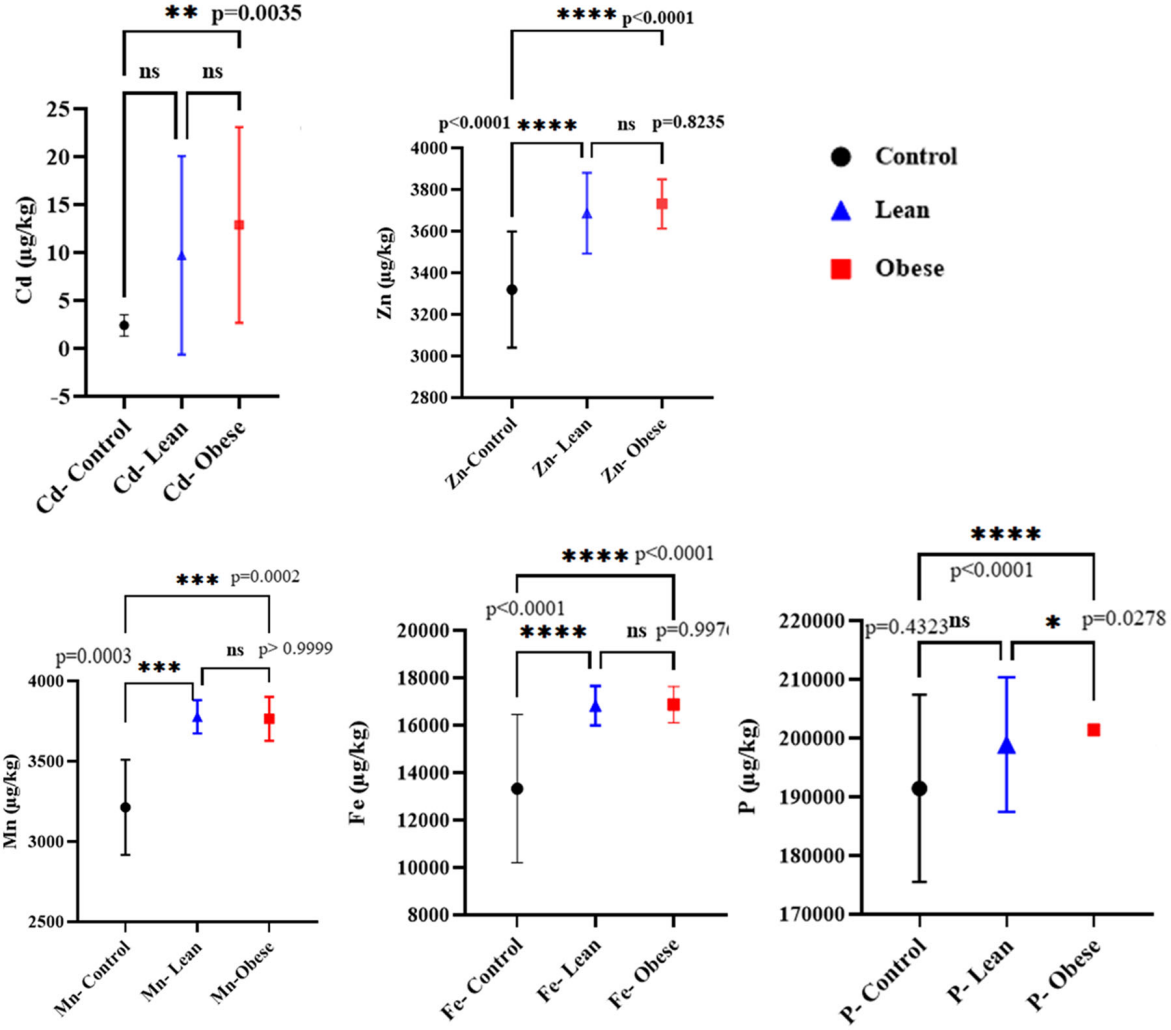
The significantly higher concentration of Zn, Cd, Fe, Mn, and P in the gut of the Lean and Obese groups.

According to the statistical analysis based on the One way ANOVA, Tukey's multiple comparisons for Parametric data, and Dunn's multiple comparisons test for non‐parametric analysis (Kruskal–Wallis test), the concentration of the Cd, Zn, Fe, Mn, and P were significantly higher in the feces samples of the individuals with abnormal weight (both Lean and Obese groups) compared to the control group with normal or healthy weight (Figure 2).

**Figure 3:**
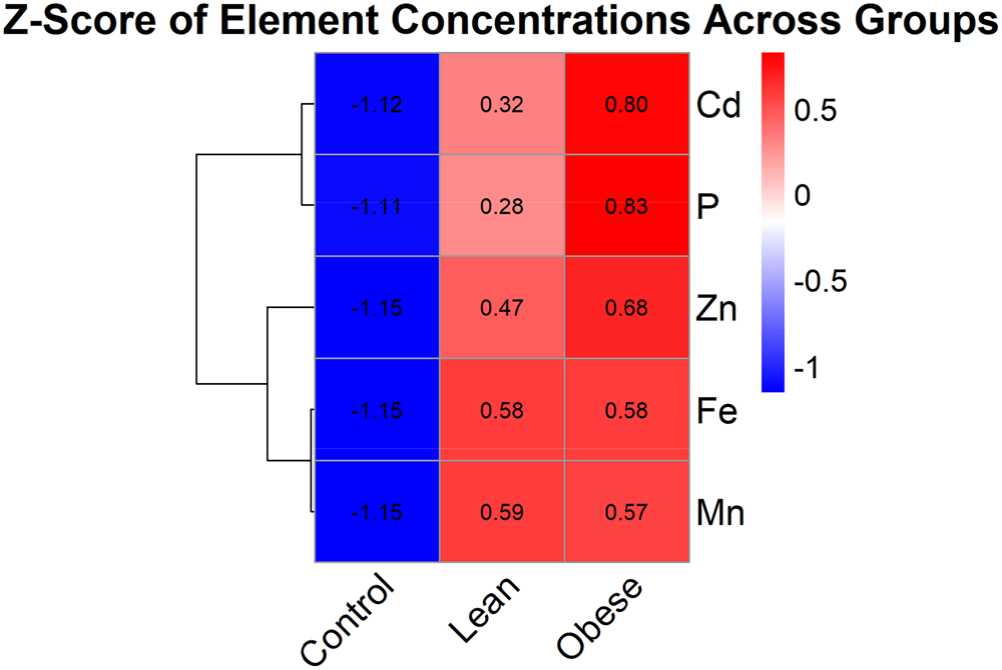
Heatmap of Z‐score normalized concentrations of significantly elevated elements in fecal samples between study groups.

To demonstrate the consistent global pattern, we have included a new **Z‐score** heatmap (Figure 3). This visualization synthesizes all data, standardizing the concentrations for each element to reveal the overall trend. The heatmap clearly shows that the Control group consistently exhibits lower concentrations (negative Z‐scores, blue) for all elements, while the Lean and Obese groups consistently exhibit higher concentrations (positive Z‐scores, red). This robustly illustrates the pattern of uniform upregulation across these metals in the gut of the Lean and Obese groups.

### 3.3 Gut Microbiota Analysis

Gut bacteria composition at the phylum, class, family, and genus level in the Obese and the control group were analyzed by CLC Microbial Genomics Module 23.0.3 and the OTUs from samples with low abundance were omitted to simplify the visualization of the OTU clustering results.

#### 3.3.1 Operational Taxonomic Units (OTU) Clustering of Gut Microbiota

The metagenomics analysis of the bacterial gut microbiota in the obese group by phylum level showed that Actinobacteria had the most abundance (1,020,588 reads) and the highest percentage 45%. The other three bacterial phyla, in order of greater abundance, include Firmicutes (965,561, 43%), Euryarchaeota (157,417, 7%), and Proteobacteria (72,337, 3%) (Supporting Information S1: Figure S1a).

In the lean group, Actinobacteria phylum had the most abundance (448,691 reads, 44%). The second rank was related to the Fimicutes phylum (406,540 reads, 40%), and the Proteobacteria with 89,762 reads was the next rank (9%) (Supporting Information S1: Figure S1b).

In the control group, Firmicutes was the first of most abundance of bacterial phyla (55%). The second rank was related to the Actinobacteria (33%). Other ranks were the phyla Euryarchaeota, Proteobacteria, and Verrucomicrobia take up 7, 3, and 2%, respectively (Supporting Information S1: Figure S1c).

**Figure 4:**
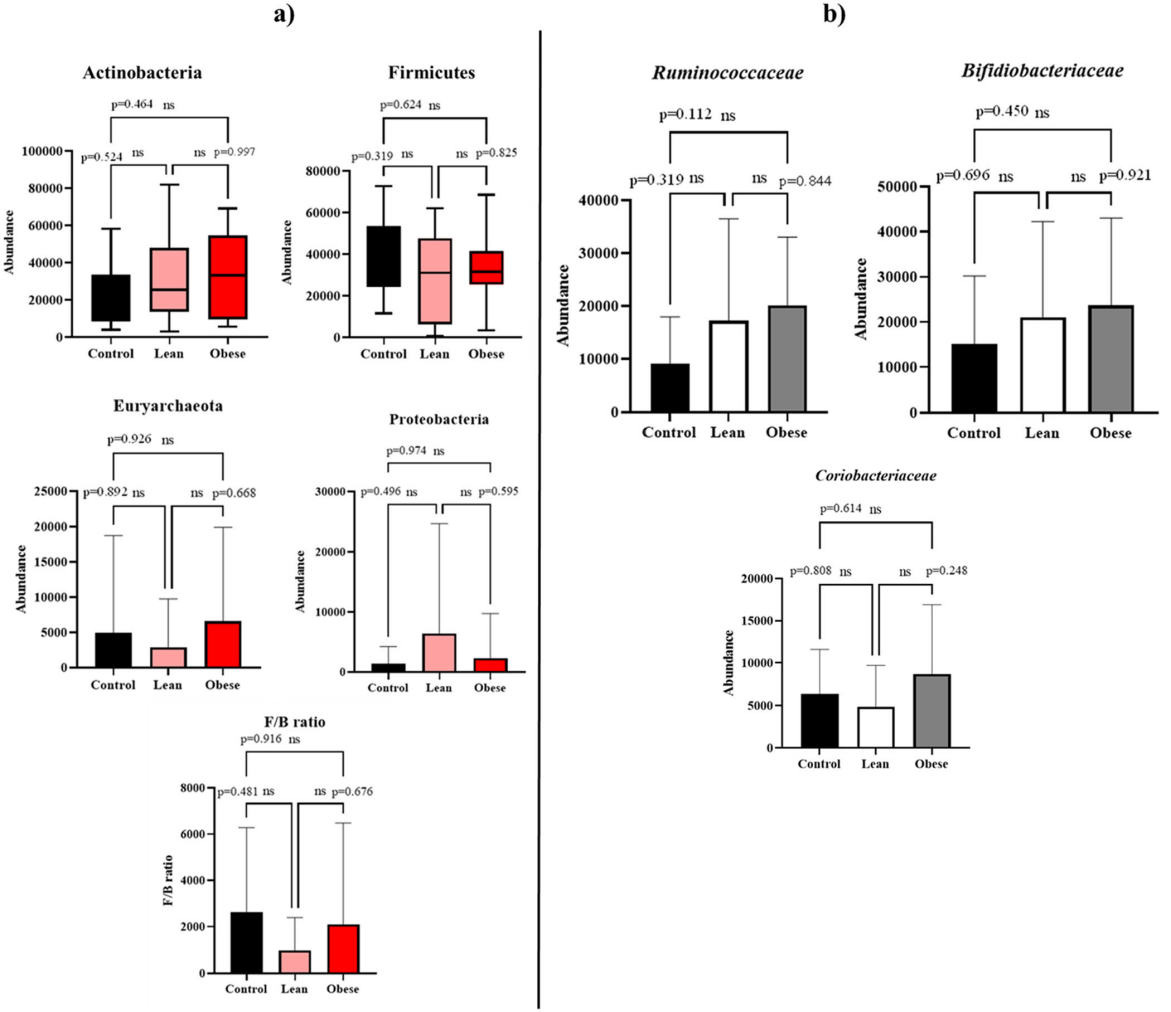
Comparing the abundance differences of the bacterial phyla (a) and bacterial families between groups (b).

Four bacterial phyla with the most abundance in three groups were selected to compare statistical differences. The statistical results revealed no significant differences in the abundance of the Actinobacteria, Firmicutes, Euryarchoaeota, and Proteobacteria phyla in the obese and lean groups compared to the control group. Also, there was no significant difference between the obese and lean groups (*p*‐values are shown in Figure 4a). In addition, the Firmicutes/Bacteroides (F/B) ratio in the three groups was analyzed. The result showed no significant differences between F/B ratios in the three groups (Figure 4a). According to the table result from OTU Clustering of Gut microbiota by the family level in the three obese, lean, and control groups, the *Bifidobacteriaceae*, *Ruminococcaceae*, and *Coriobacteriaceae* were the first, second, and third ranks of bacterial families with most abundances, respectively (Figure 4b). Regarding the statistical analysis, there were no significant differences in the abundances of three bacterial families between groups (Figure 4b).

**Figure 5:**
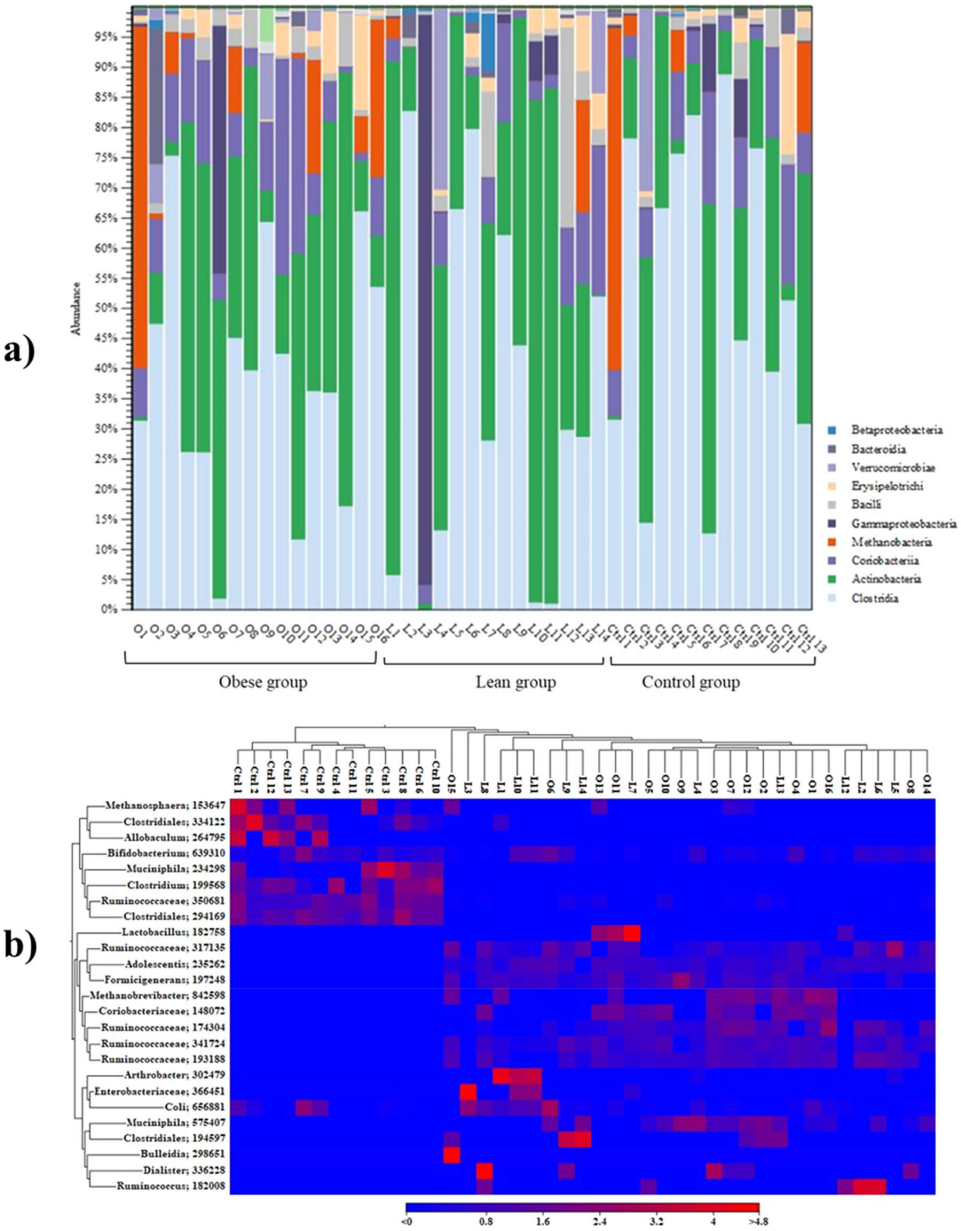
Percentage of OTU number by class level. (a) Heatmap (hierarchical clustering) for subclass level groupings gut bacteria in three groups. (b) Obese, lean, and control.

According to the OTU clustering table results, the abundance of each bacterial class in the Obese (O1–O16), Lean (L1–L14), and Control (Ctrl 1–Ctrl13) is demonstrated in the Figure 5a and was distinguished with a specific color bar. For example, in sample number one of obese individuals (O1), the most abundant gut microbiota by the class level (57%) was Methanobacteri, as shown in the dark orange bar in the Figure 5a. The second and third ranks were Clostridia (27900 reads, light blue bar) and Coriobacteria (7240, Dark purple), about 31% and 8.2%, respectively (Figure 5a).

In the Lean group, for example in the sample L1, the most abundance of the bacterial class was related to the Actinobacteria class with 78374 reads (85% green bar color), the other class were Clostridia (5362 reads, 5.8% light blue bar), Coriobacteria (3490 reads, 3.8% dark purple bar), and Methanobacteria (3106 reads, 3.45 orange bar) (Figure 5a). In the control group, for example in the Ctrl1, the Methanobacteria had 50159 reads (57% orange bar). As shown in the Fig, the light blue bar shows the Clostridia with 27,912 reads (32%). The abundance of the Coribacteria is shown with purple bar (6779 reads, 7.7%) (Figure 5a).

According to the heat map (Figure 5b), the abundance of each sub‐class in each sample is demonstrated in the obese, lean and control groups, respectively. The color intensity in each sample is normalized to define its relative ratio in the individuals. The color scale blue to red colors (< 0 – > 4.8) for the heat map is displayed at the bottom of the figure (Figure 5b). For example, in sample 1 of the obese and lean groups (O1 and L1), the *Arthrobacter* genus and the *Methanobrevibacter* had the highest abundance (red color with 2.29 and 4.1 values), respectively. In the case of the gut microbiota changes in the control samples, for example, the *Methanosphaera* and *Allobaculum* bacteria have the highest abundance in sample number 1 (Ctrl1), which is depicted with red color. In addition, the similarity of communities in each sample (O1–O16 in obese, L1–L14 in Lean, and Ctrl 1–Ctrl 13 in Control group) is demonstrated in the dendrogram (Figure 5b).

#### 3.3.2 The Overall Distribution of the Gut Microbiome With the Most Abundant Genera In the Obese, Lean, and Control Groups

**Figure 6:**
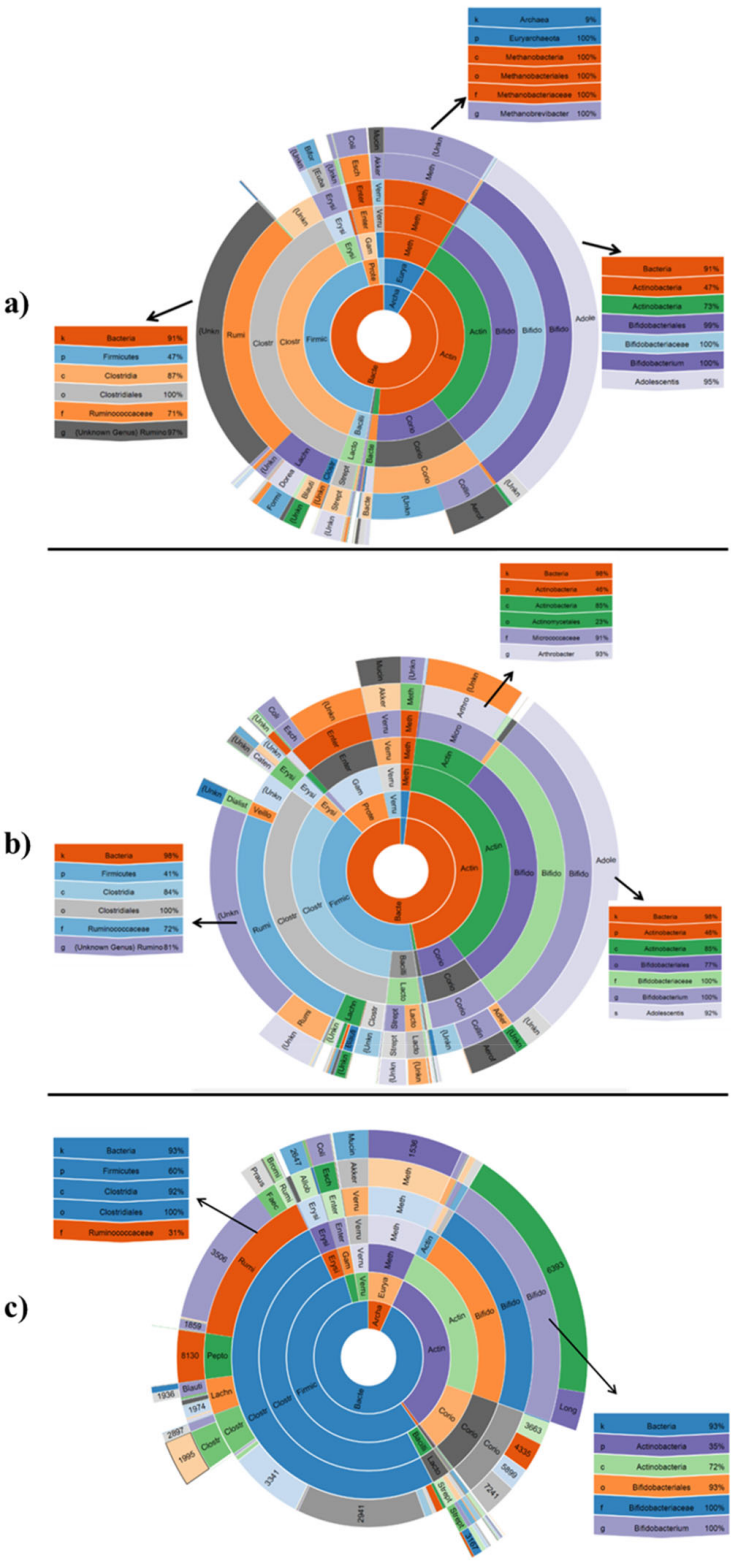
Sunburst representation of the overall distribution of the gut microbiome with the most abundant genera in the obese (a), lean (b), and control (c) groups.

In the case of sub‐family level clustering, the *Bifidobacterium. adolescents* was identified as the most abundant (359,870 reads) species of gut microbiota in the obese group. The sunburst in Figure 6a shows the taxonomy of this species. The second and third ranks of the most abundant bacterial in the gut microbiota of Obese people were Unknown Genus *Ruminococcaceae* (313,299 reads) and Unknown Species of *Methanobrevibacter* (104,943 reads). The percentage showed in the Sunburst figure depicts the percentage of the recognized bacteria by using the 16SrRNA gut metagenomic. For example in the Figure 6a, 71% of the recognized of bacteria families in the Clostridial order are belong to the *Ruminococcaceae* family. In another example, 100% of the recognized bacteria in the *Bifidobacteriaceae* family was belong to the *Bifidobacterium*. In the lean group, the *Bifidobacterium. adolescents* was the most abundant (272,491 reads) species. The second and third ranks of the most abundant bacterial were Unknown Genus *Ruminococcaceae* (193,667 reads) and Unknown Species of *Arthrobacter* (72,740 reads). The Sunburst in Figure 6b represents the overall distribution of the gut microbiome in the lean group and shows the taxonomy of these three genus. In case of the control group, the *Bifidobacterium* was the most abundant (212,538 reads) species. The next rank of the most abundant bacterial was Unknown Genus} *Ruminococcaceae* (212,538 reads).

The Sunburst in Figure 6c represents the overall distribution of the gut microbiome in the Control group.

#### 3.3.3 The Alignment Result and Phylogenetic Tree

**Figure 7:**
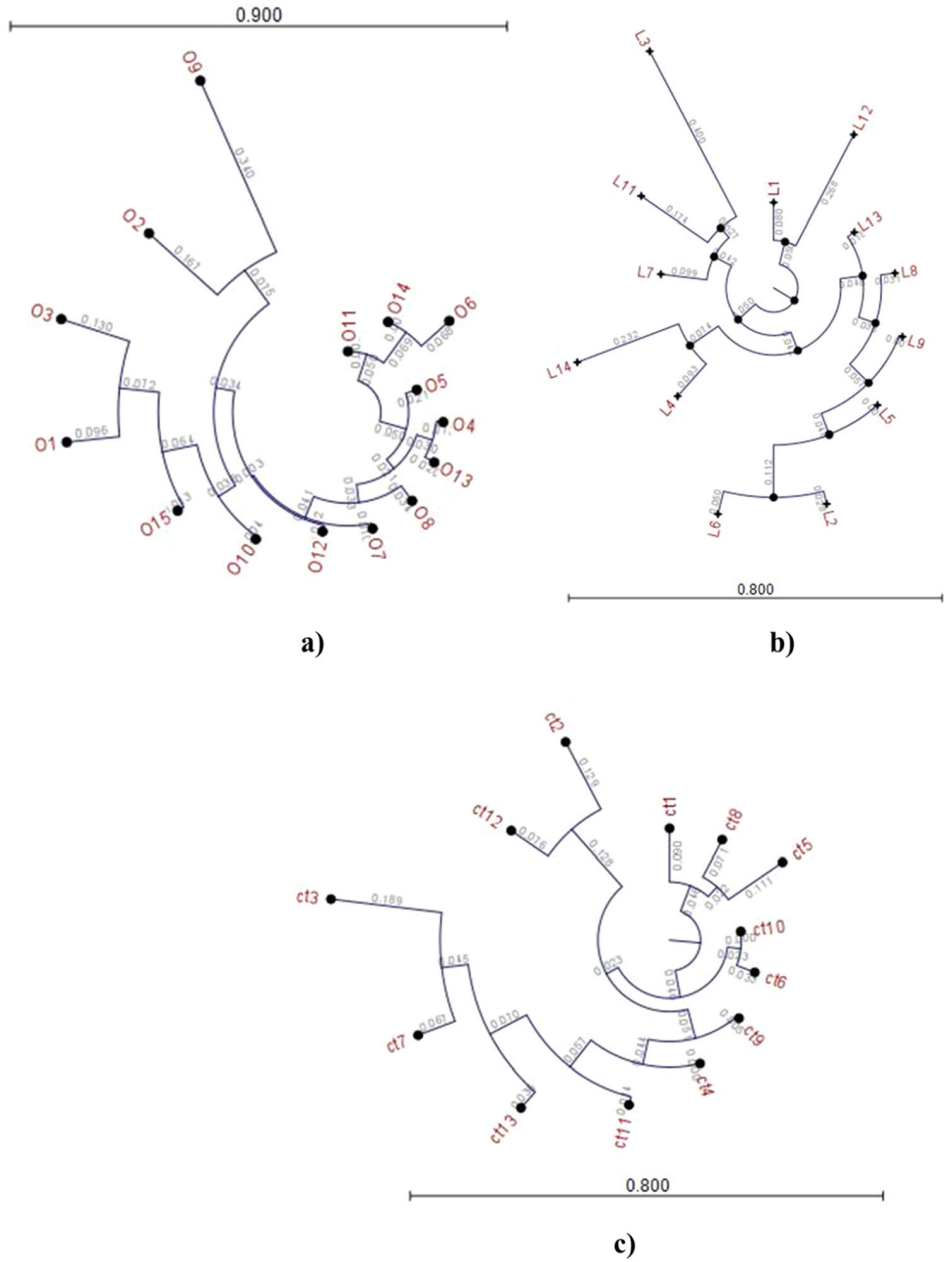
The phylogenetic tree corresponding to the gut microbiota of the obese (a), lean (b) and the control group (c).

The alignment results and corresponding phylogenetic trees for the gut microbiota of three groups are illustrated in Figure 7. The phylogenetic trees for three groups were developed using the “k‐mer model”(142). Based on the “K‐mer Based Tree Construction,” the construction of the phylogenetic trees involves using single sequences or sequence lists as input to construct a distance‐based representation of the evolutionary relationships among bacterial communities. In Figure 7a, people labeled as O1‐O16 describe members of the obese group, while in Figure 7b,c, individuals labeled as L1–L14 and C1–C13 correspond to the Lean and Control groups, respectively. The branching pattern within a phylogenetic tree depicts the evolutionary relationships among bacterial communities in obese, lean, and control individuals, indicating how species have evolved from common ancestors over time.

#### 3.3.4 Alpha Diversity and Estimation of Species Richness in Each Sample

One of the typical alpha diversity indices is the Chao Index. Chao 1 is an estimator based on abundance; therefore, it requires data that refers to the abundance of individual samples belonging to a specific class (Chao and Chiu 2016). Species richness is the count of species number or operational taxonomic units (OTUs) present in an area and is the most uncomplicated measure of alpha diversity (Thukral 2017).

**Figure 8:**
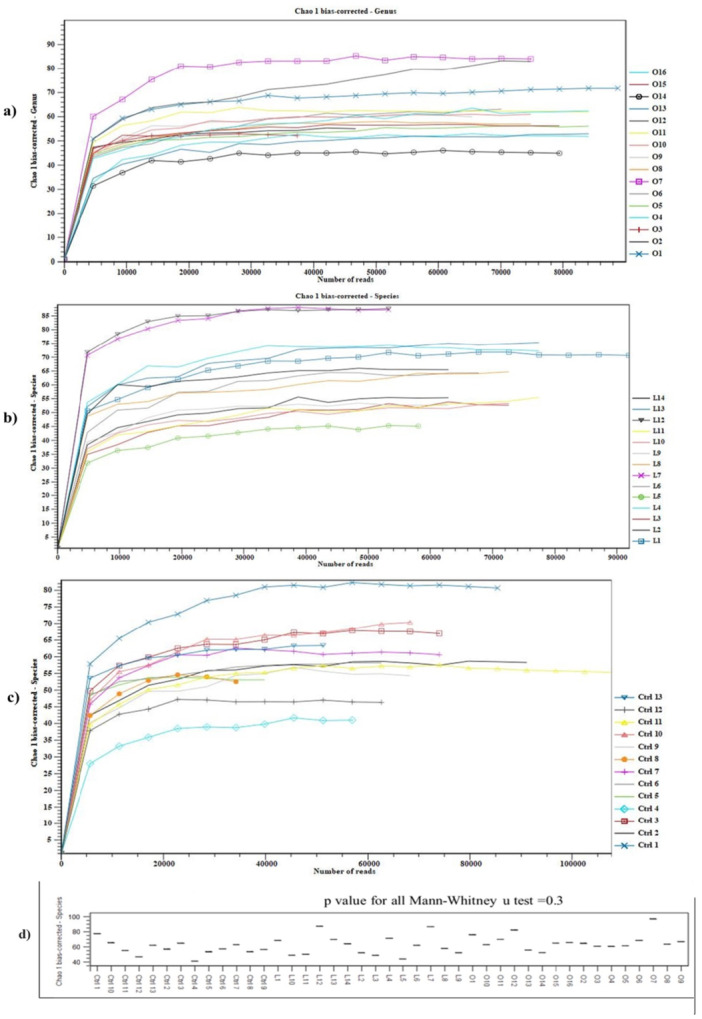
Comparing the alpha diversity (Chao 1 bias‐corrected‐ Species): obese (a), lean (b), control (c).

The number of reads in each sample number is depicted with a specific color line on the *X*‐axis. In the obese group (Figure 8a), the highest and lowest abundance numbers are related to the sample O1 (blue cross) and O3 (red plus). The highest Chao 1 index (82) is demonstrated in sample number 7 (O7 purple square). In conclusion, the larger the Chao 1 value, the greater species diversity in obese individuals. The lowest Chao1 index (40) is related to the sample number 14 (O14 black circle). In case of lean group, as shown in the *X*‐axis of Figure 8a and according to the OTU table result by species level, the highest abundance number (91,949 reads) is in sample number 1 (L1 blue square). Two sample numbers L7 (cross purple) and L12 (black reverse triangle) have lowest abundance but with highest Chao 1 indexes (approximately 85). As shown on Figure 7b, the lowest Chao 1 index (40) in the lean group is related to the L5 (green circle).

Regarding the control group, as shown in the *X*‐axis (Figure 8c), sample number 11 (Ctrl11 with yellow triangle) has the highest abundance (108151 reads according to the OTU table result). The sample number 8 (Ctrl 8 with orange circle) has the lowest abundance (39200 reads). The most species richness was related to sample number 1 (Ctrl1 with blue cross line) due to the highest Chao 1 index (approximately 82). However, the lowest index (approximately 40) was related to sample number 4 (Ctrl 4 with light blue diamond). The most Choa1 index in the obese, lean, and control groups were 82, 85, and 82, respectively (Figure 8). In addition, comparing Chao1 indexes in the *y*‐axis shows same species diversity in the three group and regarding the Mann‐whitey u test (*p* = 0.3 > 0.05) there were not any significant differences between species diversity of groups (Figure 8d).

#### 3.3.5 Beta Diversity Assessment

**Figure 9:**
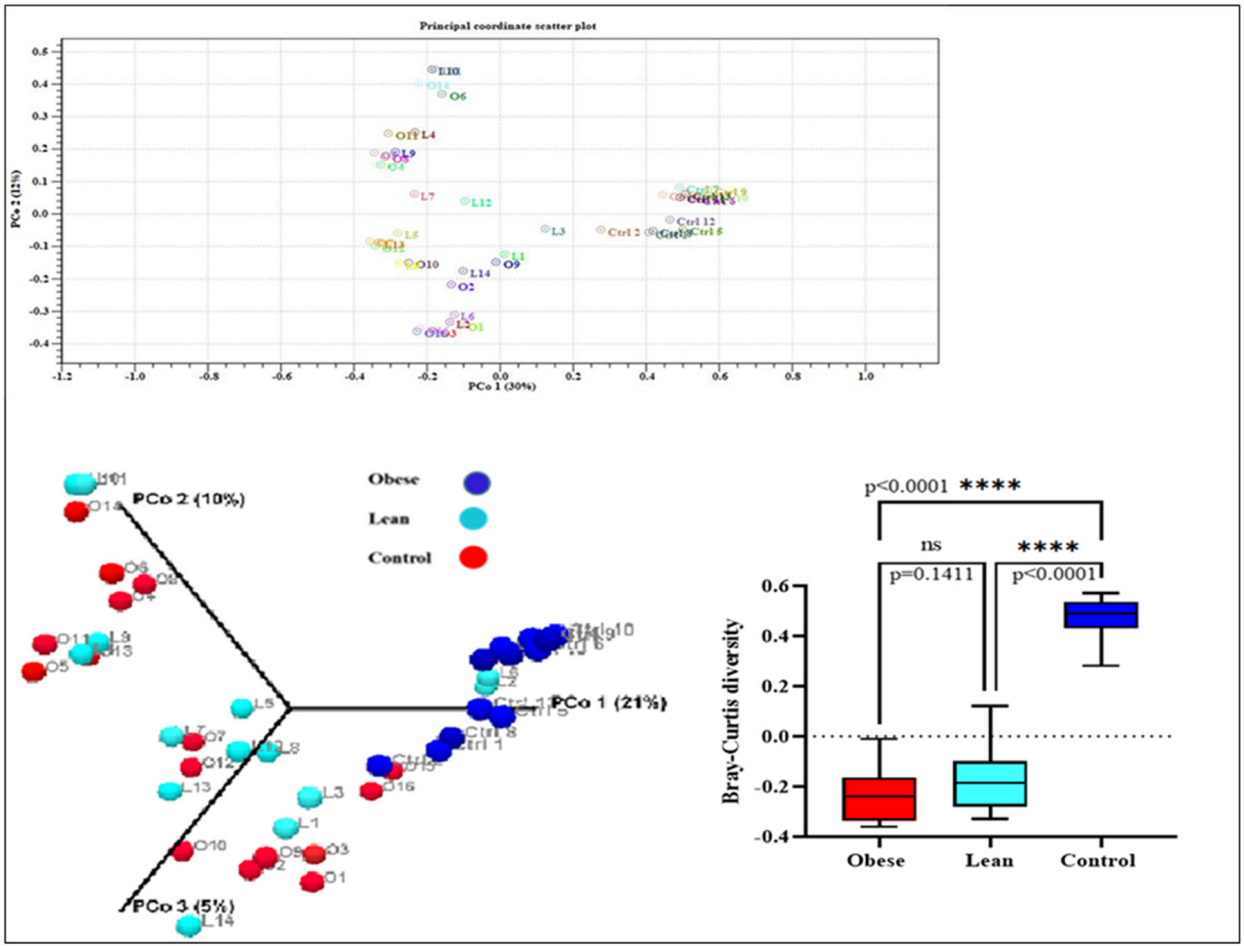
Comparing the Beta diversity (Bray‐Curtis diversity) between three groups.

Shah et al (2023) showed that Bray‐Curtis as a distance metrics estimate microbial variation among different gut samples (Shah et al. 2023). Principles Component Analysis (PCA) was used and a scatterplot was generated to determine the phylogenetic distances between bacterial communities in samples O1‐O16 (obese group), L1–L14 (Lean group), and Ctrl 1–Ctrl 13 (Control group) (Figure 9). The axes indicate the percentages of variation in the data for the bacterial communities. The red, light blue and dark blue circle symbols shows bacterial communities in the obese, lean, and control groups, respectively. As shown in the Figure 9, the statistical analysis showed significant difference of Bray‐Curtis dissimilarity in the obese and lean groups compared to the control group (*p* < 0.0001) and no significant difference was found between obese and lean group (*p* = 0.141). So, it was shown that there is more phylogenetic distance between the control group (normal BMI) with two other obese and lean groups (abnormal BMI). Supporting Information S1: Table S2 shows the the beta‐diversity analysis in all analyzed metrics: bray‐curtis dissimilarity, Jaccard index and Euclidean metrics between three groups.

#### 3.3.6 Correlation of BMI V.S Firmicutes and BMI V.S Bacterides

In this study, we examined the correlation of BMI with an abundance of Firmicutes and Bacteroides. The result from the non‐parametric Spearman correlation showed a positive correlation of BMI v,s Firmicutes, and a negative correlation of BMI v.s Bacteroides. There was no correlation between BMI and the abundance of these bacterial phyla in the lean and the control group. Supporting Information S1: Table S3 shows the Spearman *r*, 95% confidence interval, and *p*‐values of the Multiple Comparisons in the obese, lean, and control groups.

#### 3.3.7 Correlation of the Abundance of *Bifidobacteriaceae* With Cd Concentration in Fecal Samles

The significant decrease in the Cd concentration in the fecal samples of Obese individuals encouraged us to examine the Correlation of the Bifidobacteriacea abundance (The most abundant of bacteria in the obese group) with Cd concentration in fecal samples. The result showed a negative correlation of Bifidobacteriaceae abundance v.s Cd (*r* = −0.6629, CI: −0.8720 to −0.2490, *p* = 0.0051), where the abundance of the Bifidobacteriacea increase, the Cd concentration in the feces is decreased. Bifidiobacterium are involved in promoting heavy metal excretion via feces, inducing heavy metal detoxification enzymes, or remediating of heavy metal toxicity.

**Figure 10:**
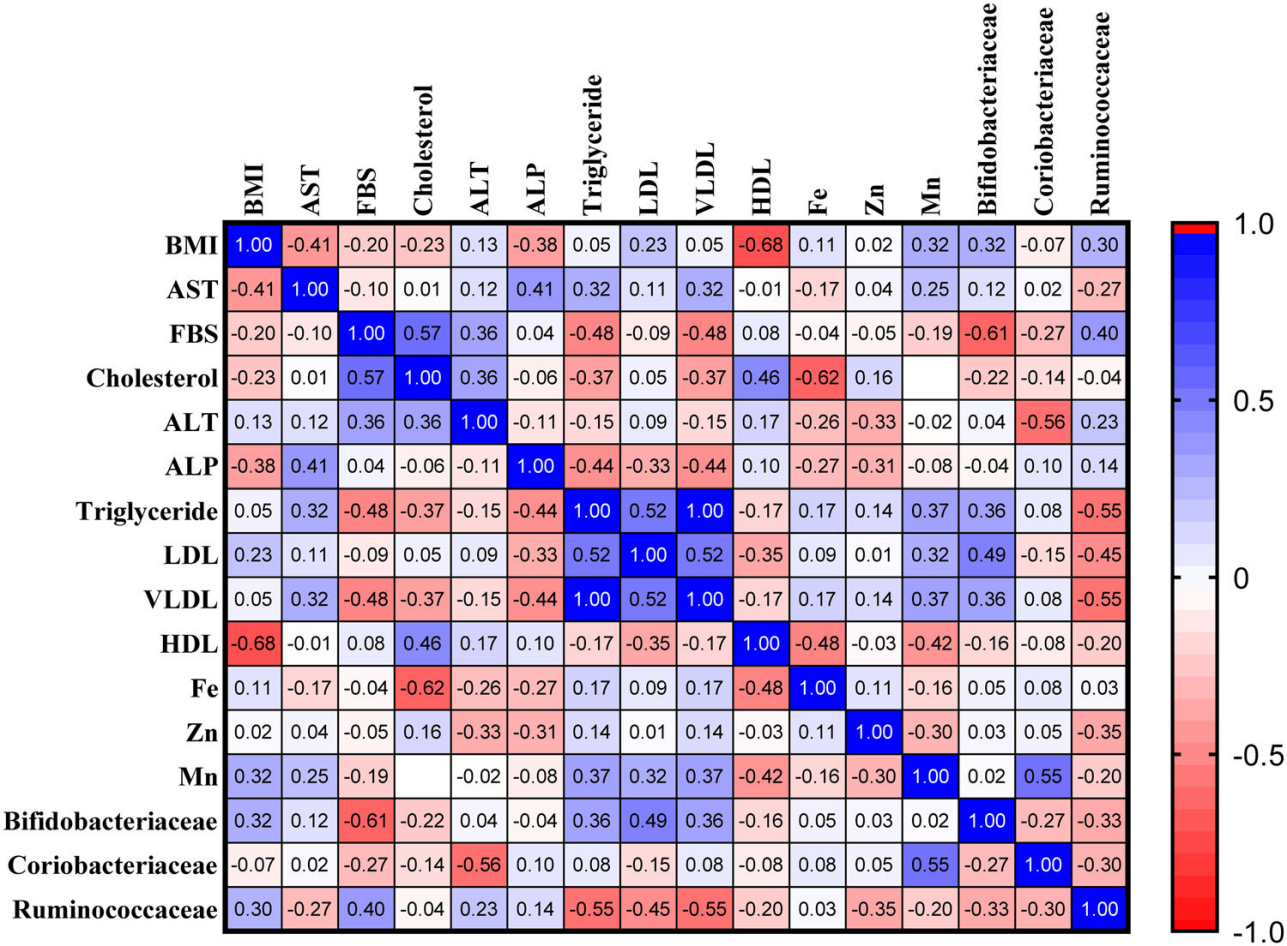
Multivariate Correlation Network in the Lean Phenotype. Correlation matrix of clinical metabolic parameters, elemental concentrations, and dominant gut microbial families in the lean group. Blue indicates positive correlations and red indicates negative correlations.

Correlation analysis of the lean phenotype revealed a number of significant correlations between gut microbiota abundance, elemental levels, and clinical variables. We observed strong negative correlation of HDL cholesterol with BMI (*r* = −0.68, *p* = 0.007), showing raised HDL with reduced body mass index in this phenotype. In addition, iron level was highly negatively correlated with total cholesterol (*r* = −0.62, *p* = 0.019). Microbial analysis revealed that abundance of *Bifidobacteriaceae* was negatively correlated with fasting blood glucose (*r* = −0.61, *p* = 0.021), while abundance of *Coriobacteriaceae* was positively correlated with manganese level (*r* = 0.55, *p* = 0.040) and ALT enzyme activity (*r* = 0.56, *p* = 0.039). Additionally, abundance of *Ruminococcaceae* was negatively correlated with triglyceride and VLDL (*r* = −0.55, *p* = 0.041) levels (Figure 10). These findings demonstrate the complex interaction between metabolic health, environmental metal exposure, and gut microbial ecology in the determination of the lean phenotype.

## 4 Discussion

### 4.1 High Glucose Level in the Obese People

Elevated fasting blood sugar (FBS) levels in obese individuals can be explained through various interconnected physiological mechanisms. It's important to note that the relationship between obesity and elevated blood sugar is complex and involves multiple factors such as insulin resistance, inflammation (Zeyda and Stulnig 2009), adipose tissue dysfunction (Blüher 2009), hormonal imbalance (Seth et al. 2013), genetic factors (Barness et al. 2007), physical inactivity (Eaton and Eaton 2017), and dietary factors (Seiler et al. 2018).

### 4.2 High Level of the Cholesterol, TG, LDL, and VLDL in the Obesity

The relationship between obesity and high cholesterol, specifically elevated low‐density lipoprotein (LDL) cholesterol, is multifaceted and involves several interconnected physiological mechanisms. Some of these mechanisms are including: insulin resistance and metabolic syndrome (Steinberger et al. 1995), dyslipidemia (Howard et al. 2003), inflammation (Fan et al. 2019), Adipokines (Derosa et al. 2013), hepatic lipid synthesis (Simonen et al. 2011), dietary factors (Calder et al. 2011), genetic factors (Rankinen et al. 2015; Youngblom et al. 2016), physical inactivity (Strasser 2013).

Derosa, et al. in 2013 confirmed the higher level of the LDL in obese compared to the nonobese people, and showed that obese subjects are predisposed to develop dysmetabolic disease and hormonal dysfunctions (Derosa et al. 2013).

The liver plays a central role in lipid metabolism. In obesity, there is often an overproduction of cholesterol and triglycerides in the liver, contributing to elevated LDL cholesterol levels (Schonfeld and Pfleger 1971). Diets high in saturated fats and trans fats, commonly associated with a Western diet and fast food, can contribute to elevated LDL cholesterol levels. Excessive calorie intake, especially from sources high in unhealthy fats and refined sugars, can contribute to obesity and dyslipidemia (Christiansen et al. 1997).

### 4.3 Higher Activity of the Liver Enzymes in the Obesity Group Compared to the Normal Individuals

The higher activity of liver enzymes in individuals with obesity compared to those with normal body weight can be explained by several interconnected physiological mechanisms. Elevated liver enzyme levels are often indicative of liver inflammation or damage. **Nonalcoholic Fatty Liver Disease (NAFLD)** is a common liver disorder associated with obesity. In NAFLD, excess fat accumulates in the liver, leading to inflammation and potential liver cell damage. The inflammatory response and cellular damage in the liver result in the release of AST and ALT into the bloodstream, reflecting increased enzyme activity (Shim et al. 2010). **Insulin resistance** can contribute to the development of NAFLD. Insulin resistance in the liver can lead to increased synthesis of triglycerides and the accumulation of fat, contributing to liver inflammation and elevated liver enzyme levels (Liu et al. 2021). **Inflammatory cytokines** released by adipose tissue and other sources can directly affect the liver, leading to increased enzyme activity. The inflammatory response in the liver may result in the release of AST and ALT as part of the immune system's reaction (Shim et al. 2010). **Excess release of free fatty acids** from adipose tissue in obesity can contribute to lipotoxicity in the liver. Lipotoxicity involves the accumulation of fat within liver cells, leading to oxidative stress and inflammation. Lipotoxicity‐induced inflammation can cause damage to liver cells, resulting in the release of AST and ALT into the bloodstream (Ao et al. 2020). **Dyslipidemia**, characterized by abnormal lipid levels in the blood, is common in obesity. Elevated levels of triglycerides and low‐density lipoprotein (LDL) cholesterol can contribute to liver dysfunction and increased liver enzyme activity (Deeb et al. 2018). **Genetic factors** may play a role in both obesity and liver enzyme abnormalities. Some individuals may be genetically predisposed to conditions like NAFLD (Sookoian and Pirola 2017).

**Alterations in the gut microbiota**, observed in obesity (Zhu et al. 2014), can influence liver function through the gut‐liver axis. Changes in gut microbial composition may contribute to liver inflammation and enzyme elevation (Lu et al. 2020).

### 4.4 The Comparison in Excretion of Heavy Metal in the Feces of the Control, Lean, and Obese Groups

The ICP analysis results indicated an elevated concentration of certain heavy metals, including Cd, Zn, Fe, and Mn, in the fecal samples of groups with abnormal weight (Lean and obese). This suggests heightened excretion of Cd, Zn, Fe, and Mn in these groups, compared to the control group. The excretion of metals in feces is influenced by various factors such as individual's health status, dietary habits, and other relevant factors leading to either an increase or decrease in concentration.

### 4.5 Correlation of the BMI and Heavy Metal With Gut Microbiota in the Obese People

The correlation result of BMI in the obese individuals with the abundance of Firmicutes (F) and Bacteroides (B) showed a negative correlation of BMI v,s Firmicutes (*r* = −0.5096, CI: 0.8082 to −0.002487, *p* = 0.0457), where the abundance of Firmicutes decreased when the BMI increased. It also demonstrated a positive correlation of BMI v.s Bacteroides (*r* = 0.5851, CI:0.1101 to 0.8425, *p* = 0.0190), where the abundance of Bacteroides increased when the BMI increased, and this finding was in line with previous study by Zeng et al. (2023). Further investigations are needed to address the hypothesis that high BMI and higher abundance of Bacteroides might be associated with decreased metabolism.

Although changes in the ratio of F/B have been noted in some studies, with a higher F/B ratio associated with obesity (Pinart et al. 2021), this study reported no significant differences in the F/B ratio of obese, lean, and control groups, which was consistent with previous studies (Hu et al. 2015; Stojanov et al. 2020). According to the negative correlation of *Bifidobacteriaceae* v.s Cd concentration (*r* = −0.6629, CI: −0.8720 to −0.2490, *p* = 0.0051), in the fecal samples of the obese people, this result was consistent with a review study by Duan et al. (2020), in which the enrichment of *Bifidobacterium* is associated with promoting heavy metal excretion via feces, inducing heavy metal detoxification enzymes, or remediating heavy metal toxicity (Duan et al. 2020).

### 4.6 Nonsignificant Differences in the Abundance of the Bacterial Communities in Three Groups

The gut microbiome is a complex ecosystem where different microbial groups interact with each other. Changes in the abundance of one bacterial family can influence the dynamics of other bacterial families. The abundance of bacterial families in the gut microbiome of obese and lean individuals compared to control individuals can be influenced by various factors.

Diet plays a crucial role in shaping the composition of the gut microbiome. Differences in dietary patterns between obese and nonobese individuals can influence the abundance of specific bacterial families. For example, certain diets high in fat and low in fiber have been associated with changes in the gut microbiome, favoring the growth of some bacterial groups. Obesity is often associated with metabolic changes, including alterations in glucose and lipid metabolism. The gut microbiome can be influenced by these metabolic changes, leading to shifts in the abundance of specific bacterial families that may play a role in metabolism (Aron‐Wisnewsky et al. 2021). Obesity is characterized by chronic low‐grade inflammation, and the gut microbiome can influence the immune system. Changes in the relative abundance of specific bacterial families may be associated with immune responses and inflammation (Tarantino 2014).

Individual genetic factors can also influence the composition of the gut microbiome. Genetic variations among individuals may contribute to differences in the abundance of specific bacterial families (Kolde et al. 2018).

Factors such as physical activity, stress levels, and antibiotic use can impact the gut microbiome. Lifestyle differences between obese and nonobese individuals may contribute to observed changes in microbial composition (Karl et al. 2018).

As mentioned in the result section there were not any significant differences in bacterial abundance among lean, obese, and control individuals. It is possible that the absence of significant differences in bacterial abundance among lean, obese, and control individuals in metagenomic results is due to the inherent biological variability within the human microbiome. Even individuals within the same category can exhibit substantial differences, so it's challenging to detect subtle variations with insufficient sample size and inadequate statistical power. The F/B ratio can vary widely among individuals, and the microbiome is known for its high interindividual variability.

The lack of significance may be due to the natural variability within each group. Additionally, various factors such as diet, physical activity, medications, and lifestyle variables can impact the microbiome. Moreover, the microbiome can exhibit temporal dynamics, so recent dietary changes or interventions may impact the results. Therefore, it's crucial to consider the timing of sample collection when interpreting the results.

Differences in sampling methods, study populations, methodologies, collection, storage, DNA extraction and next generation sequencing technologies among different research studies may contribute to variations in findings (Panek et al. 2018).

### 4.7 Non‐ Significant Alpha Diversity, but Significant Beta Diversity

Phylogenetic distance between three groups based on the Beta diversity analysis showed that the gut microbiota in the obese and lean groups with abnormal BMI was different compared to the control individuals with normal BMI. But the Alpha diversity analysis based on the Chao1 estimator showed significant difference between groups due to the same Chao1 index. So no species diversity and richness was reported in the obese and lean groups compared to the control groups.

Although no significant differences of species richness based on the alpha diversity (Chao 1 index) were found between three groups (*p* all comparisons = 0.3), but we found significant differences beta‐diversity (PERMANOVA) between the different group pairs in all analyzed metrics: bray‐curtis dissimilarity, Jaccard index, and Euclidean.

The alpha diversity (Chao 1 bias‐corrected and observed OTUs) of individuals was not significantly correlated with that of gut microbiota, Whereas the beta diversity (Bray‐Curtis dissimilarity, Jaccard and Euclidean index) of diet was associated with that of gut microbiota.

As a quick reminder, alpha diversity describes the “within‐sample” diversity concerning its richness (number of taxonomic groups). It would be suggested that the species richness within individual gut microbiota does not linearly increase with BMI index. Whereas obese and lean individuals that have abnormal BMI (more or less than normal) harbor more similar gut microbiota and indicate more phylogenetic distance between the control group (normal BMI) compared to two other obese and lean groups (abnormal BMI).

## 5 Conclusion

Our study aimed to explore the association between obesity, exposure to environmental pollutants (specifically heavy metals), and alterations in the gut microbiome. Our findings reveal significant biochemical differences between obese and control individuals, including elevated levels of FBS, cholesterol, triglycerides, LDL, VLDL, AST, ALT, ALP, and lymphocytes in the blood of obese participants. The analysis of stool samples using Inductively Coupled Plasma Mass Spectrometry (ICP‐MS) demonstrated a distinctive pattern of heavy metal concentration in obese individuals, with significantly higher levels of Cd, Zn, Fe, Mn, and P compared to the control group. This suggests a potential link between obesity and the altered presence of these heavy metals in the gastrointestinal tract. In conclusion, Biochemical analyses highlighted elevated markers in obese individuals, while heavy metal analysis in stool samples revealed distinct concentrations. Despite similarities in bacterial family abundances and species diversity across obese, lean, and control groups, beta diversity analysis unveiled notable phylogenetic differences. The study further identified correlations, including a negative association between *Bifidobacteriaceae* abundance and Cadmium concentration, and a positive correlation between BMI and Bacteroides. These findings emphasize the multifaceted nature of factors contributing to obesity and underscore the importance of investigating environmental influences on health outcomes.

## Author Contributions

**Ahmed Basim Mohamed Alamer:** writing – original draft, investigation, formal analysis, resources, software, data curation. **Majid Komijani:** writing – review and editing, visualization, validation, supervision, conceptualization, investigation, methodology. **Shahnaz Shahrjerdi:** formal analysis, data curation.

## Ethics Statement

The authors have nothing to report.

## Conflicts of Interest

The authors declare no conflicts of interest.

## Supporting information

Supplemantary fig and table.

## Data Availability

To protect participant confidentiality, the data supporting the study's results are not available for public access. They can be made available (if approved) from the corresponding author, who may consider the request if it is appropriate for research purposes.

## References

[mbo370071-bib-0001] Ao, N. , Z. Ma , J. Yang , et al. 2020. “Liraglutide Ameliorates Lipotoxicity‐Induced Inflammation Through the mTORC1 Signalling Pathway.” Peptides 133: 170375. 32771373 10.1016/j.peptides.2020.170375

[mbo370071-bib-0002] Aron‐Wisnewsky, J. , M. V. Warmbrunn , M. Nieuwdorp , and K. Clément . 2021. “Metabolism and Metabolic Disorders and the Microbiome: The Intestinal Microbiota Associated With Obesity, Lipid Metabolism, and Metabolic Health—Pathophysiology and Therapeutic Strategies.” Gastroenterology 160: 573–599. 33253685 10.1053/j.gastro.2020.10.057

[mbo370071-bib-0003] Barness, L. A. , J. M. Opitz , and E. Gilbert‐Barness . 2007. “Obesity: Genetic, Molecular, and Environmental Aspects.” American Journal of Medical Genetics, Part A 143: 3016–3034. 10.1002/ajmg.a.32035 18000969

[mbo370071-bib-0004] Bergmeyer, H. U. , M. Hørder , and R. Rej . 1986. “International Federation of Clinical Chemistry (IFCC) Scientific Committee, Analytical Section: Approved Recommendation (1985) on Ifcc Methods for the Measurement of Catalytic Concentration of Enzymes. Part 2. IFCC Method for Aspartate Aminotransferase (L‐Aspartate: 2‐oxoglutarate Aminotransferase, EC 2.6.1.1).” Journal of Clinical Chemistry and Clinical Biochemistry. Zeitschrift fur klinische Chemie und klinische Biochemie 24: 497–510. 3734712

[mbo370071-bib-0005] Blüher, M. 2009. “Adipose Tissue Dysfunction in Obesity.” Experimental and Clinical Endocrinology & Diabetes 117, no. 6: 241–250. 19358089 10.1055/s-0029-1192044

[mbo370071-bib-0006] Calder, P. C. , N. Ahluwalia , F. Brouns , et al. 2011. “Dietary Factors and Low‐Grade Inflammation in Relation to Overweight and Obesity.” British Journal of Nutrition 106: S1–S78. 10.1017/S0007114511005460 22133051

[mbo370071-bib-0007] Chao, A. , and C.‐H. Chiu . 2016. “Species Richness: Estimation and Comparison.” In Wiley StatsRef: Statistics Reference Online, 1–26. Wiley.

[mbo370071-bib-0008] Christiansen, E. , S. Schnider , B. Palmvig , E. Tauber‐Lassen , and O. Pedersen . 1997. “Intake of a Diet High in Trans Monounsaturated Fatty Acids or Saturated Fatty Acids: Effects on Postprandial Insulinemia and Glycemia In Obese Patients With Niddm.” Diabetes Care 20: 881–887. 9135961 10.2337/diacare.20.5.881

[mbo370071-bib-0009] Davis, C. D. 2016. “The Gut Microbiome and Its Role in Obesity.” Nutrition Today 51: 167–174. 27795585 10.1097/NT.0000000000000167 PMC5082693

[mbo370071-bib-0010] Deeb, A. , S. Attia , S. Mahmoud , G. Elhaj , and A. Elfatih . 2018. “Dyslipidemia and Fatty Liver Disease in Overweight and Obese Children.” Journal of Obesity 2018: 1–6. 10.1155/2018/8626818 PMC6020453 30009050

[mbo370071-bib-0011] Derosa, G. , E. Fogari , A. D'Angelo , et al. 2013. “Adipocytokine Levels in Obese and Non‐Obese Subjects: An Observational Study.” Inflammation 36: 914–920. 23467997 10.1007/s10753-013-9620-4

[mbo370071-bib-0012] Deutsche, G. 1972. “Standardisierung von Methoden zur Bestimmung Von Enzymaktivitaten in Biologischen Flussigkeiten (Recommendation of the German Society of Clinical Chemistry. Standardization of Methods for Measurement of Enzymatic Activities in Biological Fluids).” Zeitschrift für klinische Chemie und klinische Biochemie 10: 182–192.

[mbo370071-bib-0013] Duan, H. , L. Yu , F. Tian , Q. Zhai , L. Fan , and W. Chen . 2020. “Gut Microbiota: A Target for Heavy Metal Toxicity and a Probiotic Protective Strategy.” Science of the Total Environment 742: 140429. 32629250 10.1016/j.scitotenv.2020.140429

[mbo370071-bib-0014] Eaton, S. B. , and S. B. Eaton . 2017. “Physical Inactivity, Obesity, and Type 2 Diabetes: An Evolutionary Perspective.” Research Quarterly for Exercise and Sport 88: 1–8. 28129048 10.1080/02701367.2016.1268519

[mbo370071-bib-0015] Fan, J. , Y. Liu , S. Yin , et al. 2019. “Small Dense LDL Cholesterol Is Associated With Metabolic Syndrome Traits Independently of Obesity and Inflammation.” Nutrition & Metabolism 16: 7. 30679939 10.1186/s12986-019-0334-y PMC6341753

[mbo370071-bib-0016] Fan, Y. , and O. Pedersen . 2021. “Gut Microbiota in Human Metabolic Health and Disease.” Nature Reviews Microbiology 19: 55–71. 32887946 10.1038/s41579-020-0433-9

[mbo370071-bib-0017] Howard, B. V. , G. Ruotolo , and D. C. Robbins . 2003. “Obesity and Dyslipidemia.” Endocrinology and Metabolism Clinics of North America 32: 855–867. 14711065 10.1016/s0889-8529(03)00073-2

[mbo370071-bib-0018] Hu, H.‐J. , S.‐G. Park , H. B. Jang , et al. 2015. “Obesity Alters the Microbial Community Profile in Korean Adolescents.” PLoS One 10: e0134333. 26230509 10.1371/journal.pone.0134333 PMC4521691

[mbo370071-bib-0019] Karl, J. P. , A. M. Hatch , S. M. Arcidiacono , et al. 2018. “Effects of Psychological, Environmental and Physical Stressors on the Gut Microbiota.” Frontiers in Microbiology 9: 2013. 30258412 10.3389/fmicb.2018.02013 PMC6143810

[mbo370071-bib-0020] Kolde, R. , E. A. Franzosa , G. Rahnavard , et al. 2018. “Host Genetic Variation and Its Microbiome Interactions Within the Human Microbiome Project.” Genome Medicine 10: 6. 29378630 10.1186/s13073-018-0515-8 PMC5789541

[mbo370071-bib-0021] Liu, C. , M. Shao , L. Lu , C. Zhao , L. Qiu , and Z. Liu . 2021. “Obesity, Insulin Resistance and Their Interaction on Liver Enzymes.” PLoS One 16: e0249299. 33882064 10.1371/journal.pone.0249299 PMC8059853

[mbo370071-bib-0022] Lu, J. F. , M. Q. Zhu , H. Zhang , et al. 2020. “Neohesperidin Attenuates Obesity by Altering the Composition of the Gut Microbiotain High‐Fat Diet‐Fed Mice.” FASEB Journal 34: 12053–12071. 32729978 10.1096/fj.201903102RR

[mbo370071-bib-0023] Oussaada, S. M. , K. A. van Galen , M. I. Cooiman , et al. 2019. “The Pathogenesis of Obesity.” Metabolism: Clinical and Experimental 92: 26–36. 30639246 10.1016/j.metabol.2018.12.012

[mbo370071-bib-0024] Panek, M. , H. Čipčić Paljetak , A. Barešić , et al. 2018. “Methodology Challenges in Studying Human Gut Microbiota–Effects of Collection, Storage, DNA Extraction and Next Generation Sequencing Technologies.” Scientific Reports 8: 5143. 29572539 10.1038/s41598-018-23296-4 PMC5865204

[mbo370071-bib-0025] Pinart, M. , A. Dötsch , K. Schlicht , et al. 2021. “Gut Microbiome Composition in Obese and Non‐Obese Persons: A Systematic Review and Meta‐Analysis.” Nutrients 14: 12. 35010887 10.3390/nu14010012 PMC8746372

[mbo370071-bib-0026] Rankinen, T. , M. A. Sarzynski , S. Ghosh , and C. Bouchard . 2015. “Are There Genetic Paths Common to Obesity, Cardiovascular Disease Outcomes, and Cardiovascular Risk Factors?” Circulation Research 116: 909–922. 25722444 10.1161/CIRCRESAHA.116.302888 PMC4416656

[mbo370071-bib-0027] Sarwer, D. B. , M. Lavery , and J. C. Spitzer . 2012. “A Review of the Relationships Between Extreme Obesity, Quality of Life, and Sexual Function.” Obesity Surgery 22: 668–676. 22293982 10.1007/s11695-012-0588-1

[mbo370071-bib-0028] Schonfeld, G. , and B. Pfleger . 1971. “Overproduction of Very Low‐Density Lipoproteins by Livers of Genetically Obese Rats.” American Journal of Physiology‐Legacy Content 220: 1178–1181. 10.1152/ajplegacy.1971.220.5.1178 5574635

[mbo370071-bib-0029] Seiler, A. , M. A. Chen , R. L. Brown , and C. P. Fagundes . 2018. “Obesity, Dietary Factors, Nutrition, and Breast Cancer Risk.” Current Breast Cancer Reports 10: 14–27. 30662586 10.1007/s12609-018-0264-0 PMC6335046

[mbo370071-bib-0030] Seitz, V. , S. Schaper , A. Dröge , D. Lenze , M. Hummel , and S. Hennig . 2015. “A New Method to Prevent Carry‐Over Contaminations In Two‐Step Pcr Ngs Library Preparations.” Nucleic Acids Research 43: e135. 26152304 10.1093/nar/gkv694 PMC4787772

[mbo370071-bib-0031] Seth, B. , S. Arora , and R. Singh . 2013. “Association of Obesity With Hormonal Imbalance In Infertility: A Cross‐Sectional Study in North Indian Women.” Indian Journal of Clinical Biochemistry 28: 342–347. 24426235 10.1007/s12291-013-0301-8 PMC3783913

[mbo370071-bib-0032] Shah, T. , Y. Hou , J. Jiang , et al. 2023. “Comparative Analysis of the Intestinal Microbiome in *Rattus norvegicus* From Different Geographies.” Frontiers in Microbiology 14: 1283453. 38029126 10.3389/fmicb.2023.1283453 PMC10655115

[mbo370071-bib-0033] Shahi, S. K. , K. Zarei , N. V. Guseva , and A. K. Mangalam . 2019. “Microbiota Analysis Using Two‐Step PCR and Next‐Generation 16S rRNA Gene Sequencing.” JoVE (Journal of Visualized Experiments) 152:10.3791/59980. 10.3791/59980 PMC6945761 31680682

[mbo370071-bib-0034] Shim, J.‐Y. , M.‐H. Kim , H.‐D. Kim , J.‐Y. Ahn , Y.‐S. Yun , and J.‐Y. Song . 2010. “Protective Action of the Immunomodulator Ginsan Against Carbon Tetrachloride‐Induced Liver Injury via Control of Oxidative Stress and the Inflammatory Response.” Toxicology and Applied Pharmacology 242: 318–325. 19913046 10.1016/j.taap.2009.11.005

[mbo370071-bib-0035] Simonen, P. , A. Kotronen , M. Hallikainen , et al. 2011. “Cholesterol Synthesis Is Increased and Absorption Decreased in Non‐Alcoholic Fatty Liver Disease Independent of Obesity.” Journal of Hepatology 54: 153–159. 20947198 10.1016/j.jhep.2010.05.037

[mbo370071-bib-0036] Sookoian, S. , and C. J. Pirola . 2017. “Genetic Predisposition in Nonalcoholic Fatty Liver Disease.” Clinical and Molecular Hepatology 23: 1–12. 28268262 10.3350/cmh.2016.0109 PMC5381829

[mbo370071-bib-0037] Steinberger, J. , C. Moorehead , V. Katch , and A. P. Rocchini . 1995. “Relationship Between Insulin Resistance and Abnormal Lipid Profile in Obese Adolescents.” Journal of Pediatrics 126: 690–695. 7751990 10.1016/s0022-3476(95)70394-2

[mbo370071-bib-0038] Stojanov, S. , A. Berlec , and B. Štrukelj . 2020. “The Influence of Probiotics on the Firmicutes/Bacteroidetes Ratio in the Treatment of Obesity and Inflammatory Bowel Disease.” Microorganisms 8: 1715. 33139627 10.3390/microorganisms8111715 PMC7692443

[mbo370071-bib-0039] Strasser, B. 2013. “Physical Activity in Obesity and Metabolic Syndrome.” Annals of the New York Academy of Sciences 1281: 141–159. 23167451 10.1111/j.1749-6632.2012.06785.x PMC3715111

[mbo370071-bib-0040] Tang, S. , X. Yu , and C. Wu . 2016. “Comparison of the Levels of Five Heavy Metals in Human Urine and Sweat After Strenuous Exercise by ICP‐MS.” Journal of Applied Mathematics and Physics 04: 183–188.

[mbo370071-bib-0041] Tarantino, G. 2014. Gut Microbiome, Obesity‐Related Comorbidities, and Low‐Grade Chronic Inflammation, 2343–2346. Oxford University Press. 10.1210/jc.2014-2074 25003243

[mbo370071-bib-0042] Thukral, A. K. 2017. “A Review on Measurement of Alpha Diversity In Biology.” Agricultural Research Journal 54: 1.

[mbo370071-bib-0043] Tinkov, A. A. , M. Aschner , T. Ke , et al. 2021. “Adipotropic Effects of Heavy Metals and Their Potential Role In Obesity.” Faculty Reviews 10: 32. 33977285 10.12703/r/10-32 PMC8103910

[mbo370071-bib-0044] Trasande, L. , and B. Blumberg . 2018. “Endocrine Disruptors as Obesogens.” In Pediatric Obesity: Etiology, Pathogenesis and Treatment, 243–253. Springer International Publishing.

[mbo370071-bib-0046] Youngblom, E. , M. Pariani , and J. W. Knowles . 2016. Familial Hypercholesterolemia.

[mbo370071-bib-0047] Zeng, B. , L. Chen , F. Kong , et al. 2023. “Dynamic Changes of Fecal Microbiota in a Weight‐Change Model of Bama Minipigs.” Frontiers in Microbiology 14: 1239847. 37928663 10.3389/fmicb.2023.1239847 PMC10623433

[mbo370071-bib-0048] Zeyda, M. , and T. M. Stulnig . 2009. “Obesity, Inflammation, and Insulin Resistance–A Mini‐Review.” Gerontology 55: 379–386. 19365105 10.1159/000212758

[mbo370071-bib-0049] Zhang, X.‐w , Y.‐h Li , L.‐s Yang , H.‐r Li , and W.‐y Wang . 2010. “[Determination of Five Trace Elements (Pb, Cd, Se, As and Hg) In Human Whole Blood by Temperature‐Controllable Wet Digestion and ICP‐MS Technique].” Guang pu xue yu guang pu fen xi = Guang pu 30: 1972–1974. 20828012

[mbo370071-bib-0050] Zhao, F. , S. P. McGrath , and A. R. Crosland . 1994. “Comparison of Three Wet Digestion Methods for the Determination of Plant Sulphur by Inductively Coupled Plasma Atomic Emission Spectroscopy (ICP‐AES).” Communications in Soil Science and Plant Analysis 25: 407–418.

[mbo370071-bib-0051] Zhu, L. , W. Liu , R. Alkhouri , et al. 2014. “Structural Changes in the Gut Microbiome of Constipated Patients.” Physiological Genomics 46: 679–686. 25073603 10.1152/physiolgenomics.00082.2014

