# Supplementary material for: Heavy Metals, Gut Microbiota, and Biochemical Markers: Unraveling the Complexities of Obesity
Source: Microbiologyopen. 2025 Oct 21;14(5):e70071. doi: 10.1002/mbo3.70071 (PMC12540913; doi:10.1002/mbo3.70071)
Supplement: Supplementary file 1 — Supplemantary fig and table. [file MBO3-14-e70071-s001.docx]

##
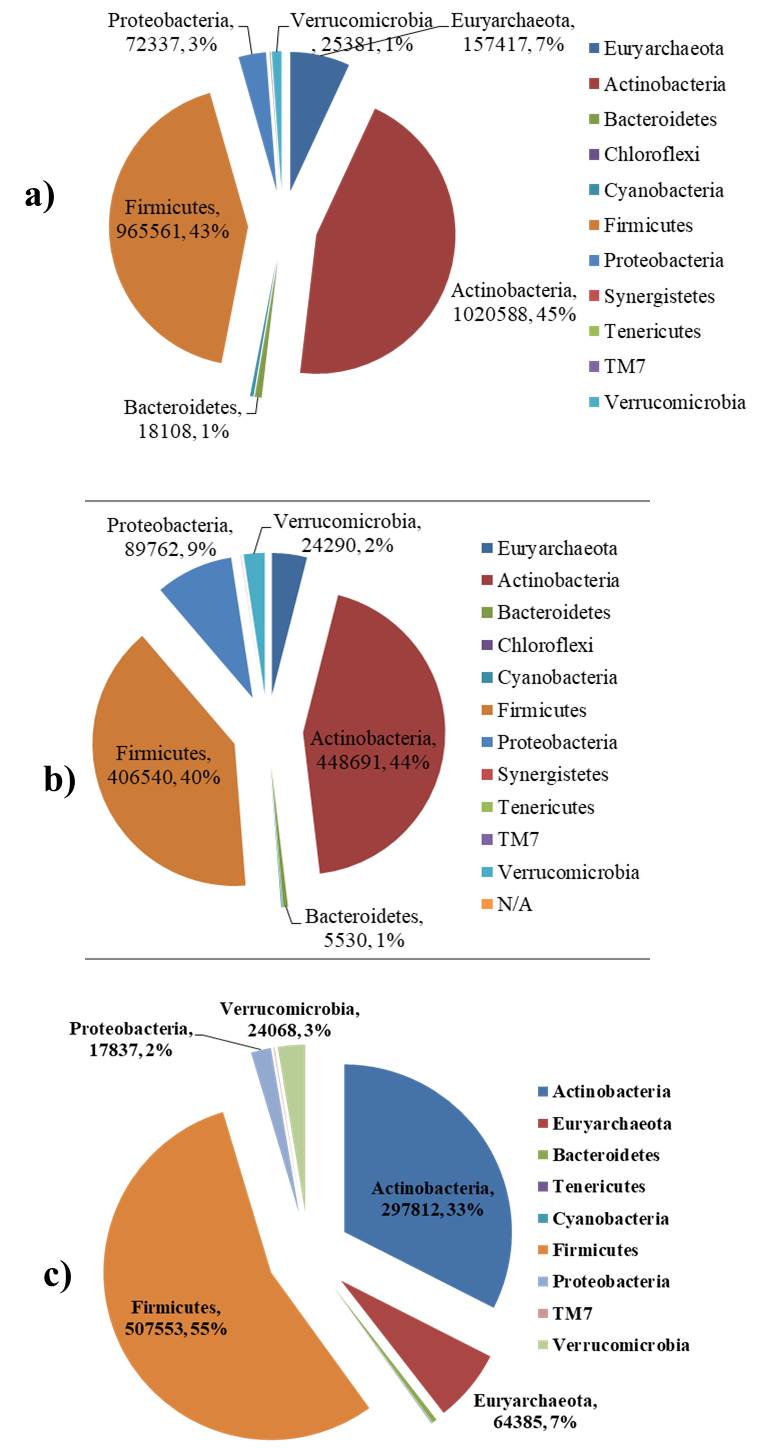


FigS1: The highest abundance of bacteria by the phylum level: a) Obese group; b) Lean and c) Control

| **Sequence** | Name |
| --- | --- |
| First Round of PCR | |
| 5'**TCGTCGGCAGCGTCAGATGTGTATAAGAGACAG**GTGYCAGCMGCCGCGGTAA3*'* | 515 F |
| 5'**GTCTCGTGGGCTCGGAGATGTGTATAAGAGACAG**CCGYCAATTYMTTTRAGTTT3*'* | 926 R |
| **Second round of PCR** |  |
| 5' **AATGATACGGCGACCACCGAGATCTACAC**CTCTCTATTCGTCGGCAGCGTC 3*'* | S502 |
| 5' **CAAGCAGAAGACGGCATACGAGAT**TCGCCTTAGTCTCGTGGGCTCGG3*'* | N701 |

Table S1: The primers used in the first and second round of PCR

Table S2: Statistical analysis for Beta diversity based on the three metrics (Bray-Curtis, Jaccard, and Eulidean index)

| **Bray-Curtis metric** |  |  |  |  |  |
| --- | --- | --- | --- | --- | --- |
| Multiple comparisons test | Mean Diff. | 95.00% CI of diff. | Below threshold? | Summary | Adjusted P Value |
| Obese vs. Lean | -0.07374 | -0.1662 to 0.01872 | No | ns | 0.1405 |
| Obese vs. Control | -0.7162 | -0.8105 to -0.6218 | Yes | **** | <0.0001 |
| Lean vs. Control | -0.6424 | -0.7398 to -0.5451 | Yes | **** | <0.0001 |
| **Jaccard Metric** |  |  |  |  |  |
| Multiple comparisons test | Mean Diff. | 95.00% CI of diff. | Below threshold? | Summary | Adjusted P Value |
| Obese vs. Lean | -0.07430 | -0.1724 to 0.02376 | No | ns | 0.1685 |
| Obese vs. Control | -0.6097 | -0.7097 to -0.5096 | Yes | **** | <0.0001 |
| Lean vs. Control | -0.5354 | -0.6386 to -0.4322 | Yes | **** | <0.0001 |
| **Euclidean metric** |  |  |  |  |  |
| Multiple comparisons test | Mean Diff. | 95.00% CI of diff. | Below threshold? | Summary | Adjusted P Value |
| Obese vs. Lean | 3586 | -9675 to 16848 | No | ns | 0.7888 |
| Obese vs. Control | 27770 | 14239 to 41301 | Yes | **** | <0.0001 |
| Lean vs. Control | 24183 | 10226 to 38141 | Yes | *** | 0.0004 |

|  | Obese BMI vs. Firmicutes | Obese BMI vs. Bacteroides | Lean BMI vs. Firmicutes | Lean BMI vs. Bacteroides | Ctrl BMI vs. Firmicutes | Ctrl BMI vs. Bacteroides |
| --- | --- | --- | --- | --- | --- | --- |
| Spearman r |  |  |  |  |  |  |
| R | -0.5096 | 0.5851 | 0.2288 | 0.2335 | 0.184 | -0.270 |
| 95% confidence interval | -0.8082 to -0.002487 | 0.1101 to 0.8425 | -0.3588 to 0.6865 | -0.3545 to 0.6891 | -0.4233 to 0.6775 | -0.7235 to 0.3463 |
| P value | 0.0457 | 0.0190 | 0.4285 | 0.4180 | 0.544 | 0.3689 |
| P value summary | * | * | Ns | ns | ns | ns |
| Significant? (alpha = 0.05) | Yes | Yes | No | No | No | No |

Table S3: The correlation of BMI v.s Firmicutes and BMI v.s Bacteroides
